# Early intervention with transitional implants for congenitally missing lateral incisors in a pediatric patient: a case report

**DOI:** 10.1186/s13256-025-05283-2

**Published:** 2025-05-19

**Authors:** Ishani Rahate, Punit Fulzele, Bhushan Mundada, Dhruvi Solanki, Nilima Thosar, Madhavi Selukar, Aakriti Chandra

**Affiliations:** 1 Department of Pediatric and Preventive Dentistry, Sharad Pawar Dental College and Hospital, Datta Meghe Institute of Higher Education and Research (Deemed to Be University), Sawangi (Meghe), Wardha, Maharashtra India; 2 Department of Oral and Maxillofacial Surgery, Sharad Pawar Dental College and Hospital, Datta Meghe Institute of Higher Education and Research (Deemed to Be University), Sawangi (Meghe), Wardha, Maharashtra India; 3 Department of Prosthodontics, Sharad Pawar Dental College and Hospital, Datta Meghe Institute of Higher Education and Research (Deemed to Be University), Sawangi (Meghe), Wardha, Maharashtra India; 4 Department of Pediatric and Preventive Dentistry, Sharad Pawar Dental College and Hospital, Datta Meghe Institute of Higher Education and Research (Deemed to Be University), Sawangi (Meghe), Wardha, Maharashtra India

**Keywords:** Transitional implant, Children, Esthetic, Pediatric prosthesis

## Abstract

**Background:**

The anterior maxillary region can be affected by traumatic or congenital loss of a tooth so that a replacement is usually essential [1]. A new innovation is the dental implantation of teeth without requiring adjustments for growth in the jaws and teeth of young patients. It is uncommon to improve the bone area surrounding the dental implant in the presence of these changes. Dental implantology is among the most innovative and fastest-growing therapeutic modes in the field of clinical dentistry. These implants are usually narrow, ranging from 1.8 to 2.5 mm in diameter, making them suitable for placement in confined spaces without affecting adjacent structures. Their insertion involves a straightforward, minimally invasive surgical procedure, often eliminating the need for significant bone modification. Composed primarily of titanium or titanium alloys, they offer excellent biocompatibility and integrate well with bone while reducing the risk of adverse biological reactions [2]. These implants have experienced significant developments over the years, through which they became a major success in the treatment of missing teeth. In this way, dentistry has transformed the way professionals work on rediscovering function, beauty, and confidence for those patients who lost their teeth through decay or severe damage. Because of progress in implant materials, techniques, and technology, dental implants have become a staple of modern restorative medicine, offering reliable outcomes and enhanced quality of life to patients of all age groups.

**Case presentation:**

A 12-year-old Indian girl came with esthetic concerns and visited the pediatric dentistry department. Orthodontic therapy was performed for the correction of malocclusion, and the missing maxillary lateral incisors were restored with MS transitional implants. The transitional implant achieved outstanding esthetic results and ensured high patient compliance.

**Conclusion:**

Transitional implants in pediatric patients offer an effective solution for managing congenitally missing laterals and preserving esthetics and function. They provide an opportunity for bone and dental development while awaiting full growth, and long-term follow-up is essential to ensure successful outcomes.

## Background

As a difficult aspect of pediatric dentistry, managing congenitally missing teeth remains a challenge. Hypodontia refers to the congenital absence of one or more permanent teeth. There is a significant impact on both the function and esthetics of a patient’s dentition when lateral incisors are missing, especially in the upper arch, where these teeth are crucial for the development of a harmonious smile and proper bite. An estimated 1–2% of maxillary lateral incisors are congenitally missing, with some reports suggesting as many as 5%. There is strong evidence that genetic factors, particularly mutations in *PAX9* and *MSX1*, are responsible for tooth size and agenesis. There is no evidence that EGF, EGFR, FGF-3, or FGF-4 are related to incisor–premolar agenesis, but signaling factors during embryonic development may play a role [1]. The anterior maxilla is thought to be the best location for implants prior to full maturity. Implant insertion has shown promising results in children as young as 5 years old because of the early closure of the symphysis joint within the first 2 years of life and the continuous growth that results in bone alterations in deposition in the face area and resorption in the dental region [2]. However, their prostheses may be adjusted to allow a 5–6 mm rise in both dental and bone height if the implants are placed closer to the front area. In these situations, a multiple-incisor prognosis is typically more suitable than a single-tooth restoration [3].

The transitional implant represents a novel and practical approach for managing missing lateral incisors in growing patients. Unlike permanent implants, transitional implants are designed to be a temporary solution. Implants do not necessitate the preparation of natural teeth, making them one of the most conservative treatment options. They are placed to maintain space, provide esthetic and functional benefits, and support the development of surrounding structures until the patient reaches skeletal maturity. Transitional implants are particularly advantageous because they help preserve bone volume and encourage natural alignment of the remaining teeth, which is essential for both function and future prosthetic rehabilitation. A key challenge in pediatric implantology lies in selecting the appropriate timing and technique for implant placement, given the potential for ongoing jaw growth [4]. Transitional implants are smaller in size, typically placed in the spaces left by missing teeth, and are often retained temporarily, offering sufficient support for prosthetic restorations without interfering with future permanent implant placement once the patient has fully developed. 

Several studies have explored the use of mini implants and transitional implants for managing hypodontia in pediatric patients. As discussed, the successful use of transitional implants in managing missing teeth in a young patient with hypodontia emphasizes the importance of individualized treatment plans [5]. Similarly, Jofré and Werner highlighted the long-term benefits of mini implants in growing children, showing that such approaches could effectively support restorative treatments without compromising the future development of the jaw [6]. This case report focuses on a pediatric patient with bilateral missing lateral incisors, managed with transitional implants to address both esthetic and functional concerns—the patient, a 12-year-old girl, presented with concerns about gaps in her smile. A careful diagnosis and treatment planning process was undertaken, considering her age, growth stage, and long-term oral health goals. This report aims to highlight the benefits, challenges, and clinical outcomes of using transitional implants in a growing patient, drawing on relevant literature and clinical experiences to underscore the potential advantages of this approach.

## Case presentation

**Fig. 1:**
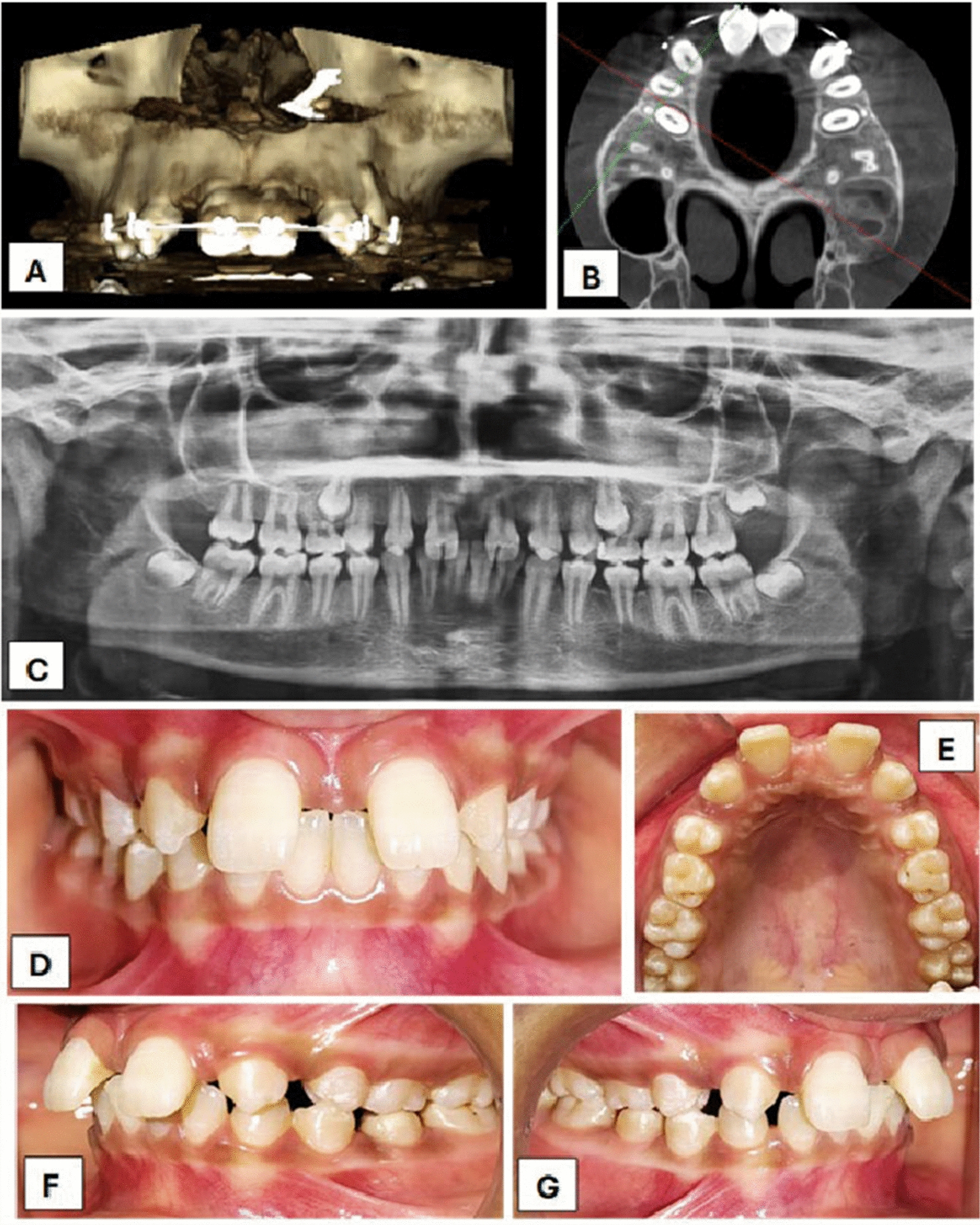
Intraoperative images **A** and **B** showing cone beam computed tomography in the mid-orthodontic phase spacing between 11 and 13, and 21 and 23. **C** Preoperative orthopantomograph. **D** Intraoral preoperative labial view. **E** Occlusal view. **F**, **G** Right and left buccal view

A 12-year-old Indian female patient in her initial clinical examination revealed a diastema and congenitally absent maxillary lateral incisors, with the canines positioned apart from the lateral incisor spaces. The patient had a straight profile, symmetrical face, competent lips, mesoproscopic facial shape, and average mouth and nose width on extraoral inspection. The patient had no relevant family history, medical history, habit history or prenatal, natal, or postnatal history. On smiling, there was upper midline diastema and spaced anterior dentition. She was concerned about her esthetic. On radiographic investigation, orthopantomography and lateral cephalogram were performed. Some elements, such as bone condition, space requirements, and bone site development, should be assessed while placing the implant. In this case, the bone density with greater width and height was present. Intra-orally, midline diastema with increased overjet and a deep bite was present (Fig. 1).

The patient also had missing teeth 12 and 22, along with hypodontia involving the maxillary premolars 14 and 24. On the lateral view, Angle’s class I was on the right side and ended on the left side, and Angle’s half class II was seen on both sides, with proclined upper anteriors. The 12-year-old girl child was diagnosed with Angle’s class I malocclusion with spacing. Clinically, the patient presented with malocclusion and esthetic deficiencies. Therefore, a multidisciplinary treatment approach was planned to restore both function and esthetics. The treatment was explained to the parents, and written informed consent was obtained from them. Treatment objectives were to correct proclined maxillary incisors, maintain a class I relationship to correct midline spacing, and replace the missing lateral incisor space with a transitional implant. Hopefully achieving a pleasing, esthetic smile and occlusion. 

### Phase 1: treatment planning

The treatment plan was made with the understanding that this situation required a comprehensive approach. The orthodontic, surgical, and restorative stages comprised the treatment plan, and the patient and her parents were informed of it.

### Phase 2: orthodontics

Transitional implants are now a standard method for replacing missing teeth, but maxillary lateral incisor implants present significant esthetic challenges. The success of the procedure, as well as the final esthetic result, largely depends on repositioning the remaining natural teeth to their correct anatomical locations [7]. Close cooperation between the restorative, implant, and orthodontic teams is necessary for this approach. Specific criteria were used to assess the orthodontic therapy, including the patient’s skeletal pattern and facial profile, the position, size, and form of the canines, the posterior occlusion, the sagittal connection between the dental arches, and the amount of space available for the incisors. The treatment strategy included correction of the midline diastema and then regaining the space needed for the implant placement of the missing maxillary right and left lateral incisors. Fixed orthodontic treatment was done to correct the diastema and alignment, as well as leveling and managing spacing in the maxillary arch. Non-extraction protocol was followed. Fixed appliance therapy with a MBT 0.022 inch bracket slot was used. Initial leveling and alignment were performed using 0.014 inch NiTi till 0.019 inch × 0.025 inch SS. Midline space closure was achieved with 0.019 inch × 0.025 inch rectangular stainless steel wires. At the mid-point of the orthodontic phase, the midline diastema was corrected along with the correction of the deep bite.

### Phase 3: surgical phase

**Fig. 2:**
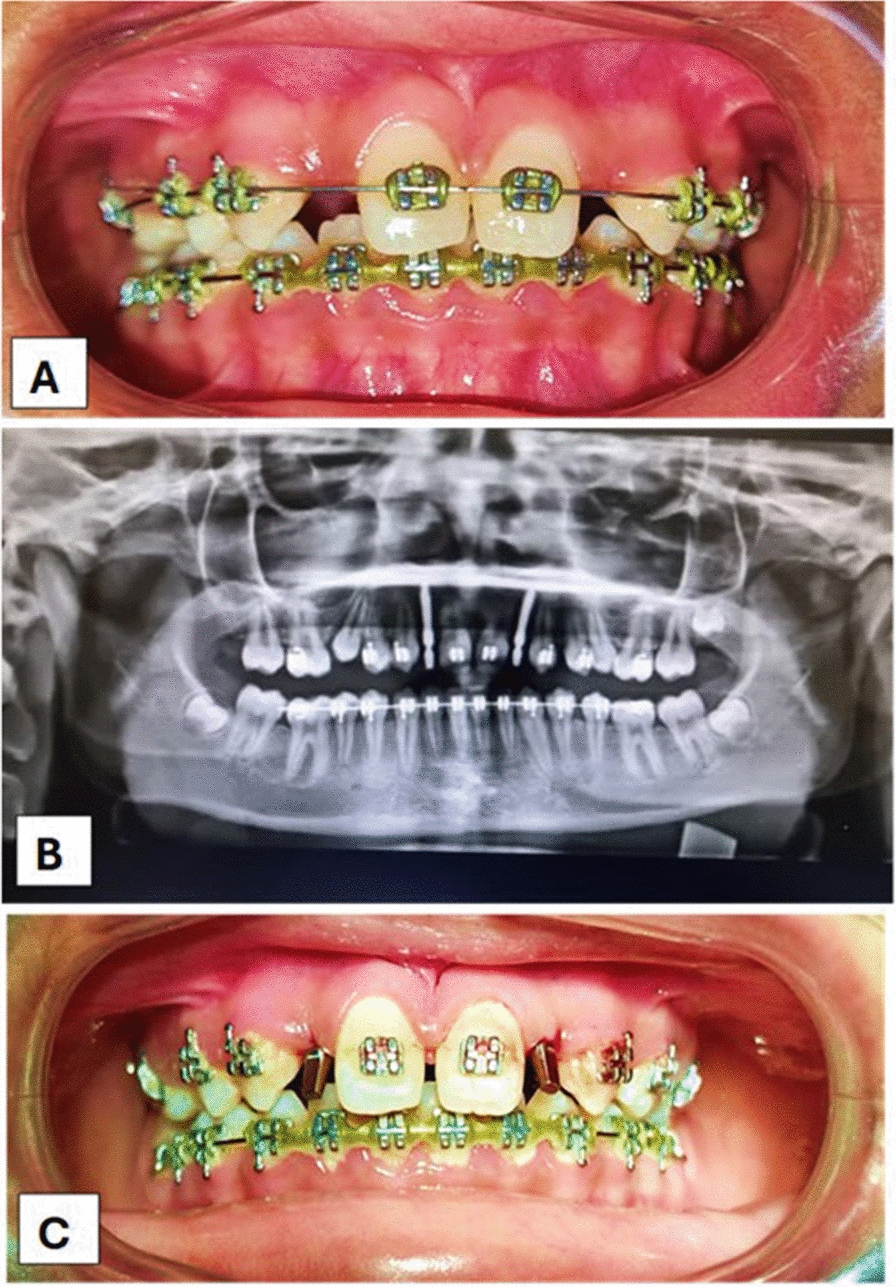
Intraoperative images. **A** Orthodontic aligning before surgery. **B** Orthopantomograph after transitional implant placement with 12 and 22. **C** T = 0, intraoral image of implant abutment immediately postoperatively

In the investigation, cone beam computed tomography was performed to evaluate bone condition. In the presurgical phase, irreversible hydrocolloid impressions of the maxillary and mandibular arches are made and poured in die stone. The bone vertical height was 13 mm and 12.1 mm of teeth 12 and 22, respectively, and the width was 3.7 and 3.8, respectively. The mesiodistal space was 6 mm for both. Further blood investigations, hemoglobin (Hb), bleeding time (BT), clotting time (CT), and random blood sugar (RBS) tests were made, and the following values were found: Hb 11 g/dL, RBS 90 mg/dL, BT 59 seconds, and CT 1.5 minutes. After all evaluations, transitional implants (Osstem) of size 2.5 mm × 13 mm were selected, as these implants are single-piece implants. No abutment was needed, and immediate loading was considered. As the blood investigations were normal, transitional implants were planned in the 12 and 22 regions. Aseptic conditions were maintained, and an infraorbital and nasopalatine nerve block, with 2% lignocaine 1:80,000 concentration adrenaline, was administered. The site was evaluated and prepared using 1.8 mm drills from the MS kit (Osstem) for the transitional implant, which had dimensions of 2.5 mm in diameter and 13 mm in length, and primary stability was attained (Fig. 2).

The implants were placed using the punch hole technique. The implant dimensions were placed in 12 and 22 regions. Postoperative instructions were given. Figure 2 shows patient preparation.

### Phase 4: restorative phase

Since it was a single implant case, an immediate impression was made of the implant site and poured with the transfer abutment in place. The impression was sent to a prosthodontic laboratory for crown prosthesis. Temporary crown placement was done in the immediate postoperative phase. The patient was recalled after a week for the final prosthesis (T = 1). Finishing and detailing of the occlusion was completed in 3 months orthodontically.

### Phase 5: follow-up

**Fig. 3:**
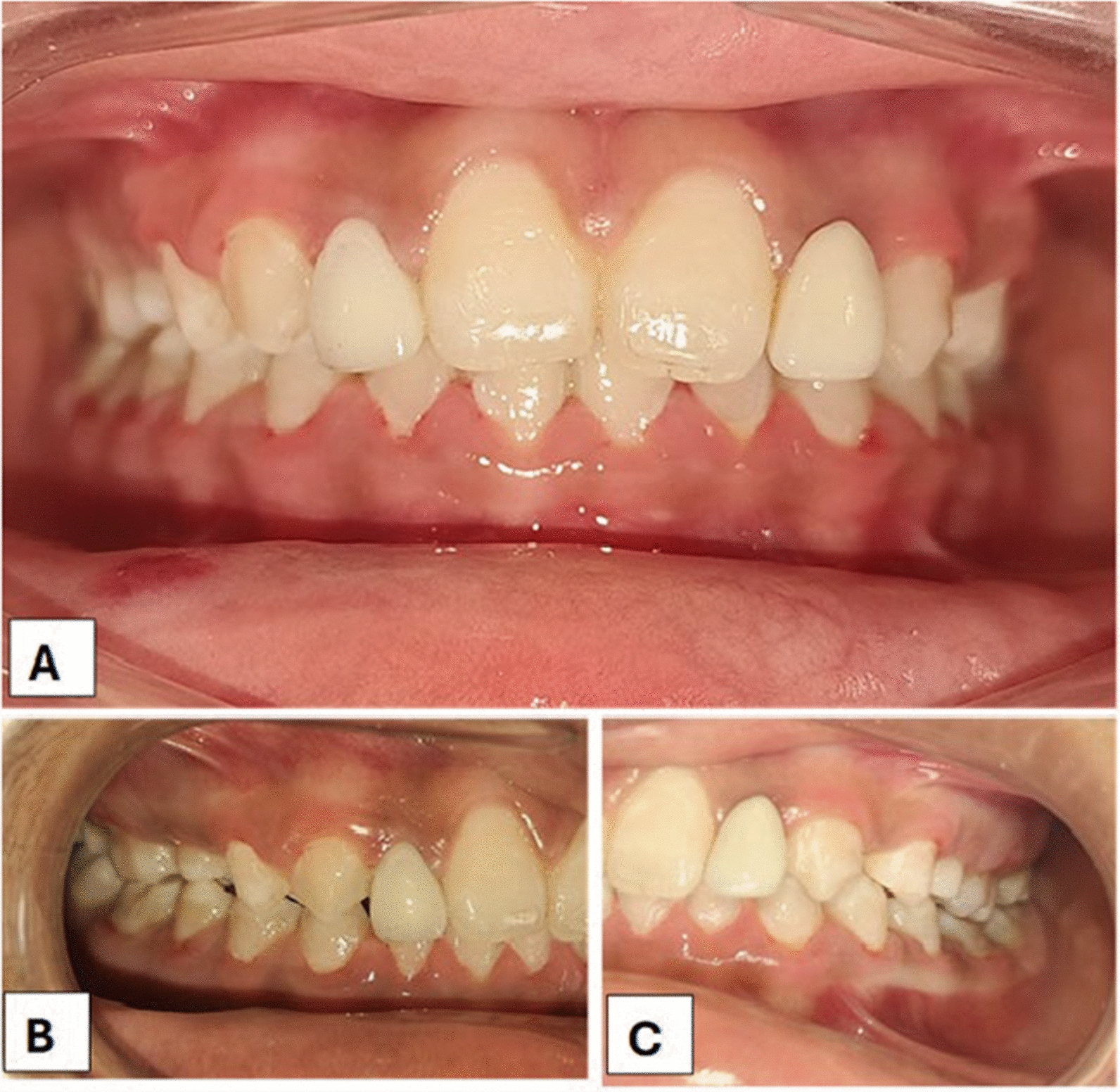
Postoperative image at 6 months (T = 2) of transitional implant of 12 and 22 with the final prosthesis.** A** Labial view,** B** Right lateral view,** C** Left lateral view

At the 3-month and 6-month follow-ups, the patient was asymptomatic, with no significant marginal bone loss or bleeding on probing during any of the visits (Fig. 3).

She will be followed up until she is 18 years old, when the definitive treatment will be started.

## Discussion

Oligodontia, also known as hypodontia, is a developmental dental defect that is sometimes linked to systemic abnormalities and disorders [8]. The most commonly affected are the second mandibular premolars, which are followed by absent maxillary laterals. The prevalence of missing teeth is somewhat higher in females than in males of the same age. The reported absence rates of maxillary lateral incisors were 10% and 18%, respectively, among Icelandic boys and girls. According to Hertzberg *et al*., 40.7% of permanent dentitions had aberrant tooth morphology, and 11.1% of individuals with Williams–Beuren syndrome (WBS) had hypodontia [9].

Students were made aware of transitional implants, their indications and applications, and their essential significance in the area of implantology. The use of transitional implants to support restorations during the osseointegration phase of definitive implants has proven to be effective, efficient, and highly beneficial. However, the most crucial element to take into account when placing implants in children and adolescents is skeletal maturation to reduce infraocclusion; this is evaluated by cephalometric analysis or hand–wrist radiography. Infraocclusion has been effectively managed with the use of a novel implant-borne prosthetic restoration [10].

The simplest alternative treatment approach in situations where the canine’s occlusion and appearance in the lateral position are acceptable is for the mesially positioned canine to close the lateral gap [1]. Both the appearance and the need for proper dental alignment should be considered when deciding the proper position of teeth next to an area where teeth are missing. In order to follow the principle of the “golden proportion” in esthetics, the gap allocated for the maxillary lateral incisor ought to be approximately two-thirds the width of the central incisor [11]. There are two forms of bone-implant interaction; Linkow depicted it as osseointegration and fibro-osseous integration. According to the American Academy of Implant Dentistry, the existence of thick, healthy collagenous tissue between the implant and bone was regarded as fibrous integration [5]. Transitional implants were designed to support temporary dental prostheses whenever needed. Simple, cost-effective, and easy to remove, they can be utilized in various stages of prosthetic rehabilitation. Typically, they are placed in a single surgical step, often without the need for incisions or sutures. The primary purpose of transitional implants is to offer retention, stability, and support for a fixed provisional prosthesis, while conventional implants undergo osseointegration [12]. Other documented uses for transitional implants include providing a fixed provisional to protect an osseous grafted site, supporting a fixed prosthetic during the healing phase, stabilizing a surgical stent during implant placement, eliminating the need for temporary tissue-borne restorations, serving as an orthodontic anchor for efficient tooth movement, stabilizing existing dentures, and replacing congenitally missing maxillary lateral incisors [3].

In this case, a transitional implant in a 12-year-old girl’s lateral incisor can be a valuable approach when there is premature tooth loss or congenital absence. At this age, the implant serves as a temporary solution to preserve space and esthetics until full development of the jaw and remaining teeth occurs. The implant supports proper alignment and prevents shifting of adjacent teeth, which could affect the future placement of a permanent prosthetic. In the present case, the patient sought treatment for esthetic concerns. After recording a detailed case history and formulating a treatment plan, fixed orthodontic brackets were placed 1 week after initial investigations to close the midline diastema and level the teeth. After 8 months, space was created for the placement of a transitional implant. A cone beam computed tomography (CBCT) scan was performed to assess the site, and implants were placed at 9 months. The final prosthesis were delivered within a week. Follow-ups were conducted at 3-month and 6-month intervals. At 14 months, the orthodontic brackets were removed. The patient will continue to be monitored until the age of 18 years.

According to Nahhas *et al*. 2014, the maxilla undergoes most of its vertical growth between ages 7 and 15 years. Transverse development primarily results from growth at the mid-palatal suture, occurring more in the posterior region than the anterior. In boys, sutural growth typically ceases around 17 years of age and in girls it ceases at 15 years. To avoid restricting maxillary transverse development, implants placed on either side of the midline should not be connected by fixed or removable restorations, either anteriorly or posteriorly, until skeletal growth is complete. Transitional implants serve as a temporary solution for growing patients, preserving space and esthetics until definitive implants can be placed after growth completion. Implants placed in late puberty or early adulthood offer a better prognosis for long-term success [13].

However, careful consideration is required to ensure the implant’s success, including monitoring bone growth and planning for future replacement with a more permanent solution once growth is complete.

## Conclusion

The use of implants in growing children offers several advantages but also raises concerns about the early placement, making it a complex issue. As there are limited studies on this subject, it is crucial for dentists to make accurate diagnoses and provide personalized treatment plans for each individual case.

## Data Availability

Not applicable.

## Abbreviations

OPG: Orthopantomograph
CBCT: Cone beam computed tomography
